# Decoding the molecular symphony: interactions between the m^6^A and p53 signaling pathways in cancer

**DOI:** 10.1093/narcan/zcae037

**Published:** 2024-09-26

**Authors:** Rachel Shoemaker, Mo-Fan Huang, Ying-Si Wu, Cheng-Shuo Huang, Dung-Fang Lee

**Affiliations:** Department of Integrative Biology and Pharmacology, McGovern Medical School, The University of Texas Health Science Center at Houston, Houston, TX 77030, USA; The University of Texas MD Anderson Cancer Center, UTHealth Houston Graduate School of Biomedical Sciences, Houston, TX 77030, USA; Department of Integrative Biology and Pharmacology, McGovern Medical School, The University of Texas Health Science Center at Houston, Houston, TX 77030, USA; The University of Texas MD Anderson Cancer Center, UTHealth Houston Graduate School of Biomedical Sciences, Houston, TX 77030, USA; Department of Integrative Biology and Pharmacology, McGovern Medical School, The University of Texas Health Science Center at Houston, Houston, TX 77030, USA; Graduate Institute of Life Sciences, National Defense Medical Center, Taipei 114, Taiwan; Department of Integrative Biology and Pharmacology, McGovern Medical School, The University of Texas Health Science Center at Houston, Houston, TX 77030, USA; Graduate Institute of Life Sciences, National Defense Medical Center, Taipei 114, Taiwan; Department of Integrative Biology and Pharmacology, McGovern Medical School, The University of Texas Health Science Center at Houston, Houston, TX 77030, USA; The University of Texas MD Anderson Cancer Center, UTHealth Houston Graduate School of Biomedical Sciences, Houston, TX 77030, USA; Center for Precision Health, School of Biomedical Informatics, The University of Texas Health Science Center at Houston, Houston, TX 77030, USA; Center for Stem Cell and Regenerative Medicine, The Brown Foundation Institute of Molecular Medicine for the Prevention of Human Diseases, The University of Texas Health Science Center at Houston, Houston, TX 77030, USA

## Abstract

The p53 tumor suppressor gene governs a multitude of complex cellular processes that are essential for anti-cancer function and whose dysregulation leads to aberrant gene transcription, activation of oncogenic signaling and cancer development. Although mutations can occur at any point in the genetic sequence, missense mutations comprise the majority of observed p53 mutations in cancers regardless of whether the mutation is germline or somatic. One biological process involved in both mutant and wild-type p53 signaling is the *N*^6^-methyladenosine (m^6^A) epitranscriptomic network, a type of post-transcriptional modification involved in over half of all eukaryotic mRNAs. Recently, a significant number of findings have demonstrated unique interactions between p53 and the m^6^A epitranscriptomic network in a variety of cancer types, shedding light on a previously uncharacterized connection that causes significant dysregulation. Cross-talk between wild-type or mutant p53 and the m^6^A readers, writers and erasers has been shown to impact cellular function and induce cancer formation by influencing various cancer hallmarks. Here, this review aims to summarize the complex interplay between the m^6^A epitranscriptome and p53 signaling pathway, highlighting its effects on tumorigenesis and other hallmarks of cancer, as well as identifying its therapeutic implications for the future.

## Graphical Abstract

**Graphical Abstract:**
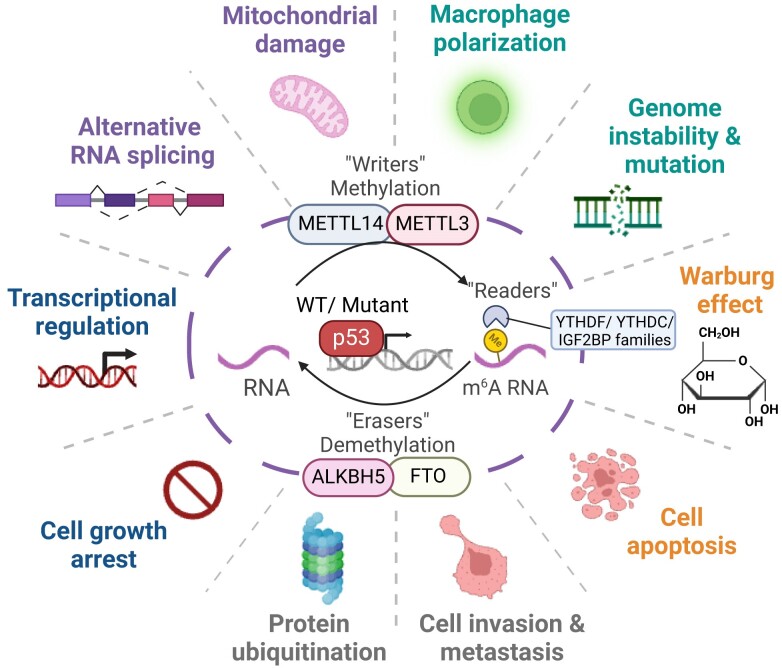

## Introduction

**Figure 1.**
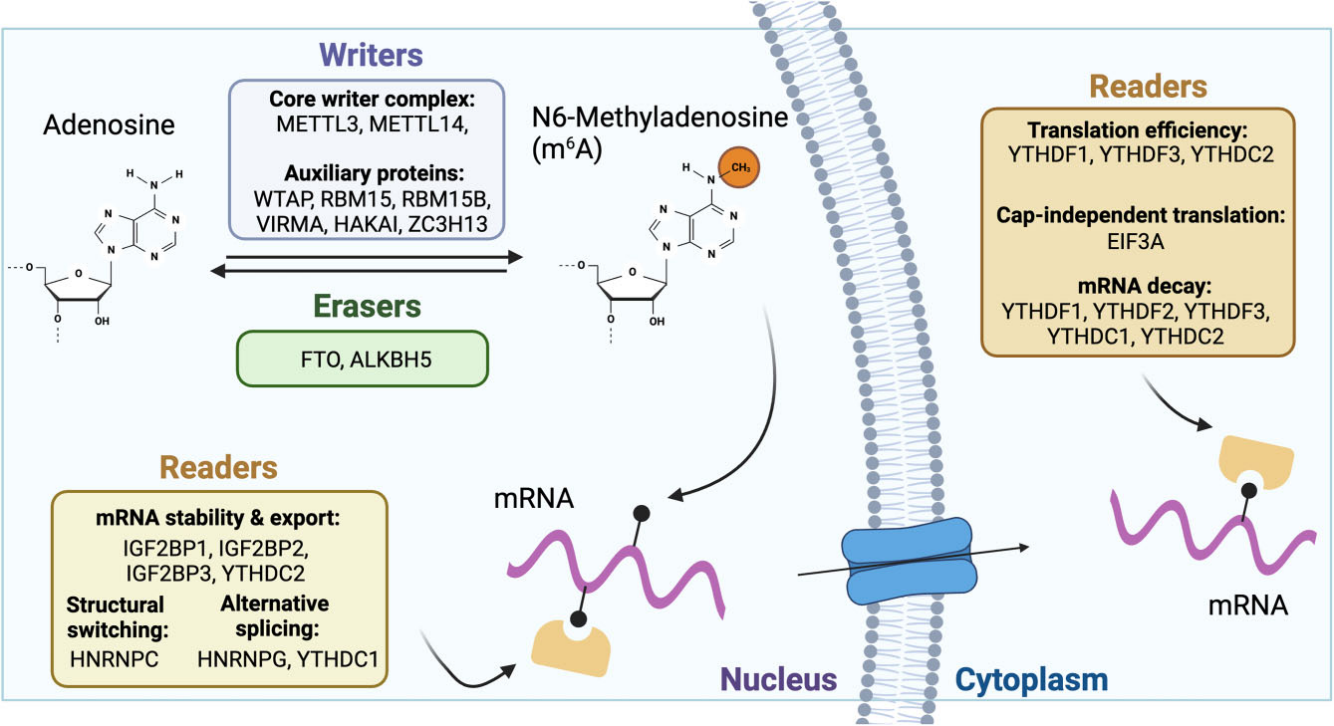
A schematic diagram illustrating key proteins in the m^6^A epitranscriptomic network. The writers (METTL14 and METTL3) and erasers (ALKBH5 and FTO) are depicted as enzymes that methylate and demethylate RNAs, respectively. The diagram highlights the biological functions of readers (IGF2BP1/2/3, YTHDC/F, EIF3A and HNRNPC/G families), which recognize and bind to m^6^A-modified RNAs. These readers influence various RNA processes, including mRNA stability and export, miRNA maturation, structural switching, alternative splicing, translation efficiency, cap-independent translation and mRNA decay. Created with BioRender.com.

Among over 150 types of chemical modifications on RNA, the *N*^6^-methyladenosine (m^6^A) modification stands out as the most prevalent, accounting for over half of all post-transcriptional mRNA modifications in eukaryotes (1). m^6^A plays a crucial role in regulating various aspects of RNA metabolism and cellular function. The dynamic process of m^6^A modification is mediated by a complex interplay of writer, reader and eraser proteins, each of which contributes to the fine-tuning of gene expression and cellular homeostasis (Figure 1) (2–5). At the core of the m^6^A modification machinery are the methyltransferase enzymes, collectively known as the writers, which are responsible for depositing methyl groups onto adenosine residues within RNA molecules. The primary m^6^A writer complex consists of METTL3 and METTL14, with METTL3 serving as the catalytic subunit and METTL14 acting as an RNA-binding adaptor (6,7). Together, these two subunits of this enzymatic complex recognize specific RNA sequences and catalyze the transfer of methyl groups from *S*-adenosylmethionine to adenosine residues, thereby generating m^6^A-modified RNA transcripts. In addition to the core writer complex, several auxiliary proteins, such as WTAP, RBM15/15B, VIRMA, HAKAI and ZC3H13, contribute to the regulation and specificity of m^6^A modification (8–10). These cofactors interact with the writer complex and modulate its activity, substrate specificity and subcellular localization, thereby influencing the patterns of m^6^A deposition across different RNA molecules and cellular contexts.

Once m^6^A modifications are deposited, they are recognized and decoded by a diverse array of reader proteins, including YTH domain-containing proteins (YTHDF1/2/3 and YTHDC1/2) and other RNA-binding proteins (EIF3, hnRNPA2B1 and hnRNPC/G). These reader proteins bind to m^6^A-modified transcripts and regulate various aspects of RNA metabolism, including mRNA stability, splicing, translation and localization, thereby exerting profound effects on gene expression and cellular phenotype (11–13). YTHDF1 and YTHDF3 are considered to promote the translation of target transcripts through the recruitment of translation initiation factors, while the reader YTHDF2 is known to promote the degradation of its target transcripts (14). However, recent investigations suggest that all YTHDF family proteins have redundant biological functions by showing similar binding to all m^6^A sites with the same primary function in mRNA degradation (15). In addition to the YTHDF proteins acting as cytoplasmic m^6^A reader proteins, YTHDC1 and YTHDC2 are nuclear m^6^A reader proteins that modulate m^6^A mRNA through distinct biological processes. YTHDC1 has been indicated to assist in the regulation of mRNA splicing and export, while YTHDC2 plays a role in mRNA translation and stability (16,17). Moreover, the dynamic nature of m^6^A modifications is further underscored by the action of eraser enzymes such as FTO and ALKBH5, which catalyze the removal of methyl groups from m^6^A-modified RNA transcripts. Through their demethylase activity, these enzymes orchestrate a complex regulatory network that governs the dynamic modification of RNA transcripts and plays a critical role in modulating gene expression, cellular differentiation and other physiological processes (7,18,19). The dysregulation of m^6^A readers, writers and erasers disrupts the expression of critical oncogenes and tumor suppressor genes through changes in m^6^A levels and RNA metabolism.

The tumor suppressor p53 is a tetrameric transcription factor well known for its role in the regulation of genes involved in a wide variety of cellular pathways (20–26). One of the most critical functions of p53 is its role in the DNA damage response. When cells experience DNA damage, p53 activates to induce the expression of genes which promote cell cycle arrest and DNA repair, leading to a halt in cell cycle progression that provides the cell with an opportunity to repair damaged DNA before replication. If the damage is irreparable, p53 can also stimulate apoptosis, effectively inhibiting the propagation of dangerous mutations. Beyond its role in the DNA damage response, p53 also regulates cellular metabolism (27,28). This gene helps coordinate an intricate network of pathways regulating glycolysis, gluconeogenesis, oxidative phosphorylation, amino acid metabolism, redox homeostasis and lipid metabolism in order to maintain cellular homeostasis and thwart the progression of diseases such as cancer, aging and metabolic disorders (29,30). Moreover, p53 can induce a state of irreversible growth arrest, known as senescence, to prevent cell proliferation (31). When p53 is activated, it can block tumorigenesis to halt cancer cell proliferation by promoting cellular senescence (32,33). Taken together, p53 has a multitude of functions that suppress tumor growth and initiation, and its diversity underscores the complexity of cellular regulation. Consequently, the loss or mutation of p53 is common in human cancers, underscoring its pivotal role in tumor suppression and the importance of understanding its role in cancer (34).

Mutations in the p53 gene are found in up to 50% of all cancers and can not only impede its function as a tumor suppressor but also transform mutant p53 into an oncoprotein with an additional gain of function contributing to tumorigenesis (35–38). While p53 mutations are identified across various domains such as the N-terminal domain (amino acids 1–97), the DNA-binding domain (amino acids 98–299) and the C-terminal domain (amino acids 300–393), they predominantly occur at one of six ‘hot-spot’ missense mutations (R175, G245, R248, R249, R273 and R282) in the DNA-binding domain, regardless of whether the mutation is somatic or germline (39). The high prevalence of these mutations suggests that p53 mutations confer a selective advantage by acquiring oncogenic functions to promote tumor initiation, progression and metastasis. One of the hallmarks of mutant p53’s gain-of-function abilities is the dysregulation of transcriptional programs, leading to the aberrant expression of genes involved in cell cycle progression, apoptotic evasion, epithelial–mesenchymal transition (EMT) and metastasis (40). Mutant p53 can bind to DNA by interacting with transcription factors, coactivators and corepressors to modulate gene expression, thereby promoting oncogenic transformation and tumor progression. Furthermore, mutant p53 engages in diverse interactions with various cellular proteins, forming novel protein–protein interaction complexes which hijack critical signaling pathways. By aberrantly modulating these cellular networks, mutant p53 fuels tumor growth and progression, creating a permissive microenvironment for cancer cell survival and dissemination, which contributes to therapy resistance (38).

Recent evidence suggests that both wild-type and mutant p53 can engage in cross-talk with the m^6^A epitranscriptomic network. Notably, dysregulation of m^6^A methylation contributes to tumor initiation, progression, metastasis and therapy resistance (41–44). In this review article, we will explore these recent findings and provide in-depth insights into how these two important signaling networks regulate each other's functions.

## Interactions between the m^6^A epitranscriptomic network and p53 signaling

### Modulating p53 signaling and tumorigenesis through m^6^A writers

The p53 tumor suppressor responds to a wide range of cellular stressors, including DNA damage, to activate anti-proliferative gene expression. In a recent study, Raj *et al.* unveiled novel mechanisms governing p53-mediated gene expression by focusing on protein–protein interactions which identified METTL3 as a novel p53-interacting protein (45). Using tandem affinity purification and mass spectrometry, they showed that METTL3 interacts with p53 through the p53 N-terminal transcriptional activation domain. Moreover, the functional interplay between METTL3 and p53 has been highlighted through data from the Cancer Dependency Map (DepMap), a comprehensive resource profiling hundreds of cancer cell lines for sensitivity to genetic perturbations using RNA interference (RNAi) or clustered regularly interspaced short palindromic repeats (CRISPR), providing Achilles scores that reflect gene essentiality for proliferation (46). Based on similar scores across cell lines, METTL3 is highly co-dependent with p53 and other positive regulators of the p53 pathway, such as TP53BP1 and ATM. Conversely, METTL3 negatively correlates with MDM2, an E3 ubiquitin ligase which negatively regulates p53 stability.

**Figure 2.**
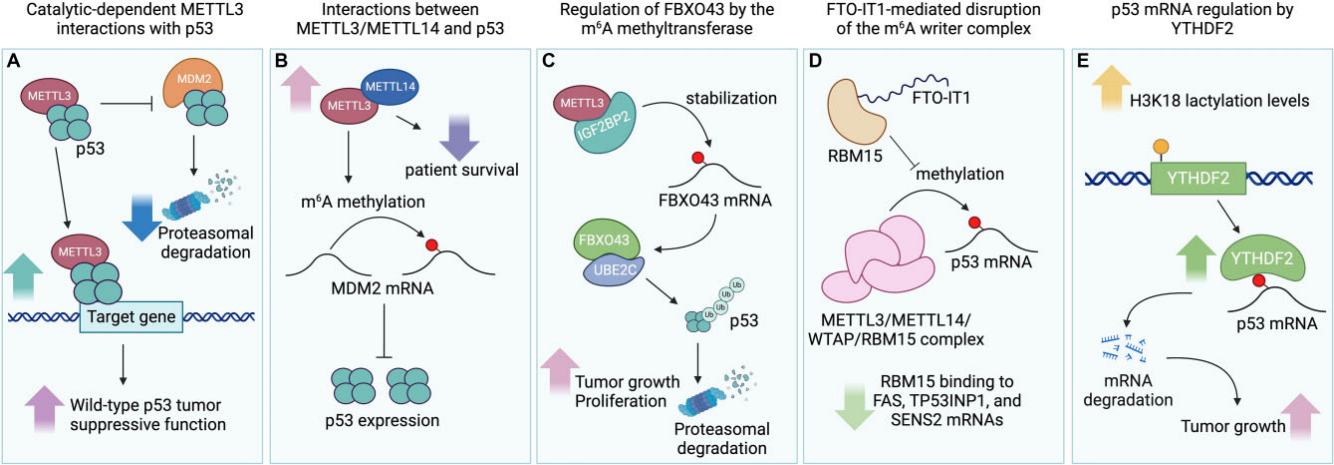
m^6^A epitranscriptomic regulation of p53 pathway signaling. (**A**) METTL3 interacts with p53 to competitively inhibit MDM2 binding and decrease proteasomal degradation. This interaction also promotes p53-mediated gene transcription, enhancing its tumor-suppressive functions. (**B**) Increased METTL3–METTL14 interactions diminish patient survival and increase m^6^A methylation of *MDM2* mRNA, which inhibits p53 expression. (**C**) METTL3 can interact with IGF2BP2 to stabilize *FBXO43* mRNA, promoting the FBXO43–UBEC interaction to ubiquitinate p53 and marking it for degradation, resulting in increased tumor growth and proliferation. (**D**) The lncRNA FTO-IT1 can interact with RBM15 to inhibit methylation of the *p53* mRNA by the m^6^A epitranscriptomic writer complex, resulting in a decrease in RBM15 interactions with the *FAS*, *TP53INP1* and *SENS2* mRNAs. (**E**) Increased H3K18 lactylation levels up-regulate YTHDF2 expression, promote *p53* mRNA degradation and enhance tumor growth. Created with BioRender.com.

Mechanistically, METTL3 stabilizes p53 by competing with MDM2, thereby preventing p53 degradation via the ubiquitin–proteasome pathway (47–49). Significantly, both wild-type and catalytic mutant METTL3 enhance p53’s transcriptional activity and prolong its protein half-life, suggesting that METTL3 activates p53 function in both catalytically dependent and independent manners. METTL3–p53 interaction maximizes p53’s response to DNA damage through increased m^6^A modification of p53 target genes after DNA damage. These transcripts modified by METTL3 are primarily involved in the mitotic cell cycle, RNA processing, cellular response to DNA damage stimulus and p53-dependent transcriptional regulation (Figure 2A). Consistent with this finding, Dominissini *et al.* demonstrated that knockdown of METTL3 in p53 wild-type cells significantly affects both the expression and alternative splicing of genes in the p53 signaling pathway (3). Notably, the MDMX isoforms, which are necessary for p53 inactivation (50), were found to be down-regulated. Together, these findings collectively underscore the crucial role of METTL3 in regulating both p53 and the p53 signaling cascade, indicating that METTL3 regulates the p53 signaling pathway by stabilizing p53 via a catalytically independent mechanism and controlling molecules involved in the p53 signaling pathway via a catalytically dependent mechanism.

### Manipulation of p53 pathway molecules by the m^6^A writer complexes

#### MDM2

Current research has illuminated the pivotal role of RNA m^6^A methyltransferase complex components, particularly METTL3 and METTL14, in the pathogenesis and progression of cancers, including acute myeloid leukemia (AML) (51) and hepatocellular carcinoma (HCC) (52). Research by Sang *et al.* revealed that the heightened expression level of METTL3 and METTL14 correlates with decreased AML patient survival and identifies the oncogenic potential of METTL3 and METTL14 in AML through the MDM2/p53 signaling axis (51). Consistently, METTL3 was shown to promote chemoresistance by enabling AML homing and engraftment (53). Knockdown of METTL3 and METTL4 inhibits AML proliferation while promoting apoptosis and differentiation. Moreover, METTL3 and METTL14 knockdown resulted in reduced m^6^A modification of *MDM2* mRNA, diminished stability and expression of MDM2, and increased p53 expression levels (Figure 2B). This regulation was also observed in studies of METTL3 function in acute kidney injury (54). Together, these findings highlight the therapeutic potential of targeting METTL3 and METTL14 in AML through modulation of the MDM2/p53 axis. In concordance with these discoveries, Yankova *et al.* revealed that METTL3 inhibition using the pharmacological inhibitor STM2457 led to decreased engraftment and increased survival in AML mice (55). Notably, STM2457 exhibited specificity in targeting AML stem cell subpopulations, suggesting its potential efficacy against treatment-resistant AML. These findings collectively advocate for the exploration of METTL3 inhibition to reactivate p53 function as a promising therapeutic strategy for AML, offering hope for improved outcomes in patients with this challenging malignancy.

#### FBXO43

Furthermore, the p53 ubiquitin ligase F-box protein 43 (FBXO43), traditionally associated with meiotic arrest, has emerged as a novel factor in the progression of HCC (52,56). Recent findings by Zhou *et al.* have identified a pivotal role for m^6^A-mediated FBXO43 stabilization in promoting HCC proliferation and invasion by facilitating p53 degradation (57). METTL3 and the m^6^A reader IGF2BP2 were shown to modulate m^6^A modification and stability of *FBXO43* mRNA, thus up-regulating its expression in HCC. Up-regulated FBXO43 facilitates the ubiquitin-dependent proteasomal degradation of p53 through interactions with UBE2C, a ubiquitin-conjugating enzyme that degrades p53. This degradation of p53 inhibits its tumor-suppressive function, creating an environment conducive to tumorigenesis (Figure 2C). Indeed, FBXO43 was identified as a marker of poor prognosis in HCC progression. Depletion of FBXO43 led to significant inhibition of cell proliferation and invasion, thus emphasizing its oncogenic potential and role in HCC progression and metastasis.

#### FTO-IT1

Additionally, the long non-coding RNA (lncRNA) FTO-IT1, transcribed from the *FTO* gene locus, has emerged as a significant contributor to prostate cancer (PCa), demonstrating overexpression during PCa progression (58). Notably, elevated levels of FTO-IT1 correlate with poor survival in PCa patients with wild-type p53 expression. Zhang *et al.* revealed that FTO-IT1 knockout enhances mRNA m^6^A methylation of select p53 target genes, thereby inducing PCa cell cycle arrest and apoptosis (58). Mechanistically, FTO-IT1 exerts its regulatory effects by directly binding to RBM15, a pivotal regulatory subunit in the m^6^A methyltransferase complex. This interaction inhibits the METTL3–METTL14–WTAP–RBM15 complex-mediated m^6^A methylation on p53 target mRNAs, thereby destabilizing these mRNAs. In cells with low or absent FTO-IT1 expression, RBM15 activates p53 tumor suppressor signaling by enhancing the stability of *FAS*, *TP53INP1* and *SENS2* mRNAs (Figure 2D). FTO-IT1 knockdown resulted in an increased binding between RBM15 and *FAS*, *TP53INP1* and *SENS2* mRNAs, whereas FTO-IT1 overexpression resulted in decreased binding between RBM15 and these transcripts. These interactions did not affect FTO-IT1-unaffected RBM15 binding targets such as MYC and the RBM15-unbound *HPRT1* transcript. Consequently, therapeutic depletion of FTO-IT1 restores mRNA m^6^A levels and expression of p53 target genes, leading to the inhibition of PCa growth in mouse models. Together, FTO-IT1 emerges as a critical regulator of m^6^A writer activity through negatively regulating p53 signaling in PCa progression, offering promising avenues for therapeutic intervention for PCa.

#### HSPA9

HSPA9, also known as Mortalin, is a member of the HSP70 chaperone protein family. It exhibits widespread expression in various cancerous tissues and cells, with elevated levels found in plasma exosomes of cancer patients. HSPA9 has been shown to bind to p53, leading to its cytoplasmic sequestration and inactivation (59). While METTL3 is known to positively regulate p53 (45), Ao *et al.* demonstrated that METTL3 negatively regulates p53 function through a paracrine mechanism in cervical cancer (60). HSPA9 expression is regulated by METTL3 through m^6^A methylation, which increases *HSPA9* mRNA stability and enhances protein translation. Exosomal HSPA9 is up-regulated in plasma exosomes isolated from cervical cancer patients. These HSPA9-enriched exosomes can impair cellular senescence and promote malignant transformation by impeding the nuclear translocation of p53 and hampering the p53–GADD45A interaction, culminating in p53 inactivation.

### Regulation of *p53* mRNA degradation and transcriptional elongation by the m^6^A reader

Recent investigations have revealed that the m^6^A reader YTHDF2 negatively regulates *p53* mRNA stability by recognizing and promoting the degradation of m^6^A-modified *p53* mRNA (61,62). Histone lactylation, a type of histone modification associated with metabolic stress, plays a crucial role in regulating gene expression in a variety of biological and pathological processes. Notably, during the Warburg effect, histone lactylation at H3K18 (H3K18la) is stimulated to induce gene expression as a response to the production of lactate under hypoxic conditions (63). Analysis of H3K18la chromatin immunoprecipitation (ChIP-seq) data, tumor-enriched transcriptomes and glycolysis-suppressed transcriptomes by Yu *et al.* revealed that the m^6^A reader YTHDF2 is a downstream target of H3K18la. Its expression is enriched in tumor cells and down-regulated upon the inhibition of glycolysis (61). YTHDF2 functions as a crucial oncoprotein in histone lactylation-induced tumor progression by recognizing m^6^A-modified *p53* mRNA and promoting its degradation (Figure 2E). Consistently, p53 down-regulation was observed when histone lactylation was induced by sodium lactate. Providing additional evidence for *p53* mRNA regulation by YTHDF2, Cai *et al.* highlighted the pivotal role of the m^6^A reader YTHDF2 in macrophage polarization, a key factor in tumor progression (62). Displaying heterogeneity and adaptability, macrophages can polarize into M1 (classically activated) or M2 (alternatively activated) phenotypes in response to different stimuli. YTHDF2, a known modulator of inflammatory gene levels during M1/M2 macrophage polarization, was found to enhance M1 polarization while inhibiting M2 polarization upon knockdown. Mechanistically, YTHDF2 knockdown up-regulated p53 by increasing *p53* mRNA stability to influence M1/M2 macrophage polarization. Together, these mechanistic insights highlight the intricate regulatory role of YTHDF2 in influencing various biological and pathological processes, such as tumorigenesis (61) and macrophage polarization (62), by down-regulating p53.

Interestingly, the recent study by Elvira-Blázquez *et al.* indicates that, instead of its m^6^A-dependent roles in splicing regulation and RNA export, the m^6^A reader YTHDC1 has an m^6^A-independent function in regulating p53 (64). In response to DNA damage, YTHDC1 directly regulates p53 transcription by binding to the transcription start sites of p53, thereby modulating the promoter-proximal pausing release of RNA polymerase II to facilitate elongation. Together, these findings highlight the versatile functions of m^6^A readers in regulating p53 expression through both m^6^A-dependent and m^6^A-independent mechanisms.

### p53-mediated transcriptional regulation of the m^6^A epitranscriptomic network

**Figure 3.**
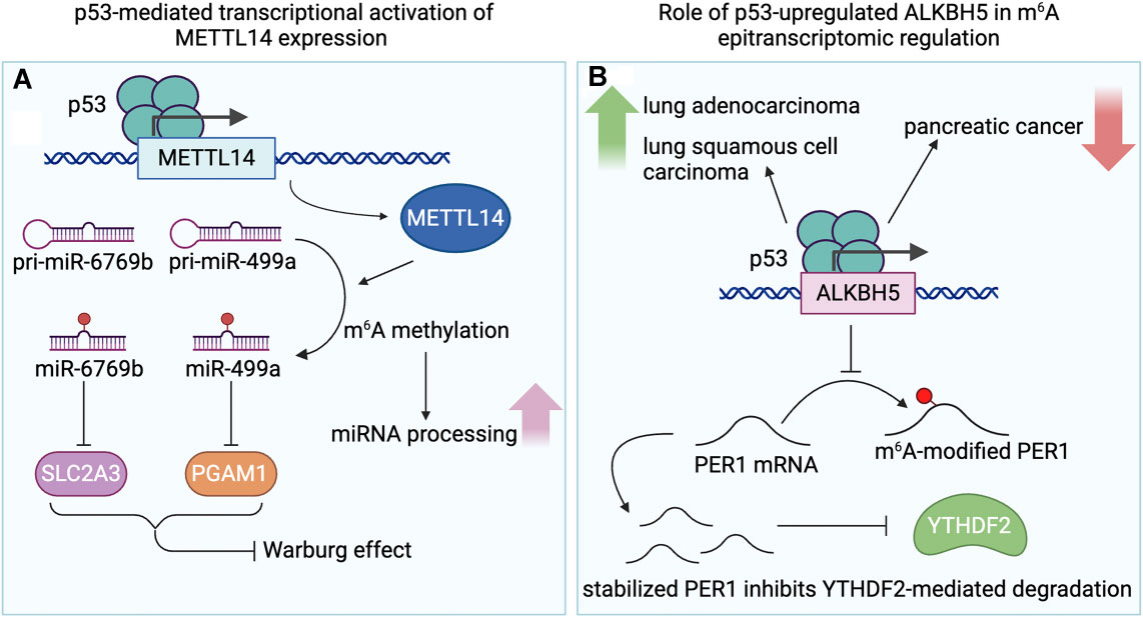
Effects of p53 signaling on the m^6^A epitranscriptomic network. (**A**) p53 binds to the METTL14 promoter to induce its expression. METTL14 can then facilitate the methylation of pri-miR-6769b and pri-miR-499a, which can inhibit SLC2A3 and PGAM1, respectively, thus inhibiting the Warburg effect. Increased methylation additionally promotes miRNA processing. (**B**) p53 regulation of ALKBH5 is increased in lung adenocarcinoma and lung squamous cell carcinoma, but it is decreased in pancreatic cancer. This interaction is known to inhibit the methylation of *PER1* mRNA. Increased levels of unmethylated *PER1* mRNA can go on to inhibit YTHDF2-mediated degradation. Created with BioRender.com.

One emerging hallmark of cancer is metabolic reprogramming, in which cancer cells exhibit altered glucose metabolism, demonstrating a preference for aerobic glycolysis even in the presence of oxygen through a phenomenon known as the Warburg effect (65). p53 plays a crucial role in repressing this phenomenon by directly and indirectly regulating key metabolic enzymes such as TIGAR and Parkin (66,67). Hou *et al.* presented an additional mechanism to illustrate how p53 modulates the Warburg effect through METTL14-mediated m^6^A epitranscriptomic regulation in colorectal cancer (68). Their investigation revealed that wild-type p53 transcriptionally activates METTL14 expression. METTL14 can counter tumorigenesis in wild-type p53 cells via the attenuation of aerobic glycolysis. Mechanistically, METTL14-mediated m^6^A modification on pri-miR-6769b/pri-miR-499a promotes microRNA (miRNA) processing and maturation, resulting in the repression of SLC2A3 and PGAM1 expression through the miR-6769b-3p/SLC2A3 and miR-499a-3p/PGAM1 axes (Figure 3A). Clinically, low METTL14 expression was found to correlate with poor prognosis in colorectal cancer, especially in p53 wild-type colorectal cancers, highlighting the relevance of the METTL14, miR-6769b-3p/SLC2A3 and miR-499a-3p/PGAM1 pathways.

p53 has also been found to transcriptionally regulate ALKBH5, an eraser protein in the m^6^A epitranscriptomic network. Research by Guo *et al.* demonstrated that p53 binds the promoter of *ALKBH5* to transcriptionally up-regulate its expression and induce post-transcriptional activation of the tumor suppressor PER1 by reducing m^6^A-modified *PER1* mRNAs. This reduction allows non-modified *PER1* mRNAs to be stabilized and prevents YTHDF2-mediated degradation. On the contrary, Liu *et al.* demonstrated that p53 can transcriptionally regulate ALKBH5 in cancer stem-like cells (69). Knockdown of p53 and inhibition of its activity by PFT-α led to the down-regulation of *ALKBH5* mRNA. Surprisingly, the malignancies of cancer stem-like cells decreased upon knockdown of p53 or ALKBH5, or inhibition of p53. A clinically relevant correlation between ALKBH5 and p53 was identified in lung adenocarcinoma and lung squamous cell carcinoma. p53-up-regulated ALKBH5 expression was shown to suppress tumor growth and invasion in pancreatic cancer (Figure 3B) (70).Together, these results highlight the complexity of p53-mediated m^6^A epitranscriptomic regulation in tumorigenesis across different cancers.

## Interactions between the m^6^A epitranscriptomic network and mutant p53 signaling

### Mutant p53-mediated neoplastic transformation through m^6^A epitranscriptomic rewiring

**Figure 4.**
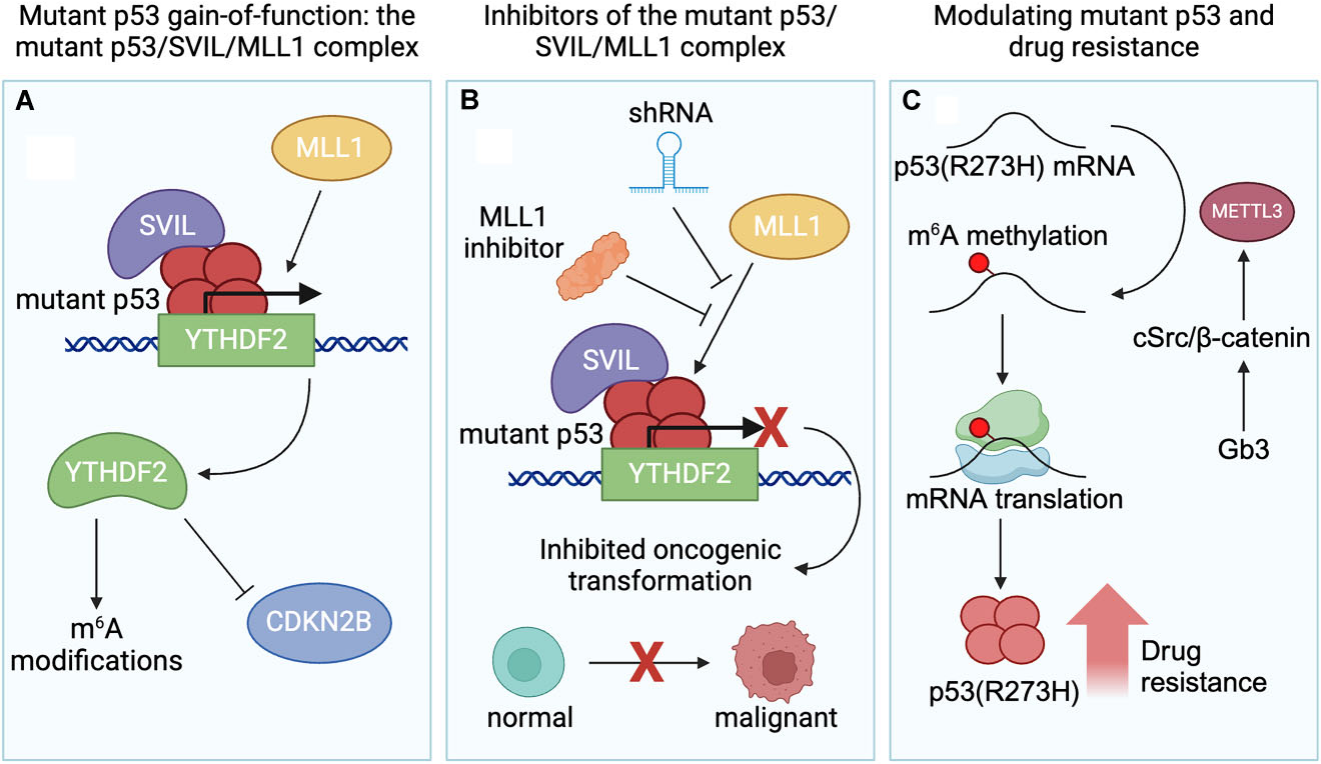
Cross-talk between mutant p53 signaling and the m^6^A epitranscriptomic network. (**A**) Mutant p53 binds to the YTHDF2 promoter and recruits SVIL and MLL1 to form a complex which results in increased levels of m^6^A modifications and decreased levels of CDKN2B. (**B**) The mutant p53–SVIL–MLL1 protein complex can be inhibited using MLL1 shRNA or inhibitors, which inhibit transcription of YTHDF2 and attenuate oncogenic transformation. (**C**) The mutant *p53(R273H)* mRNA is methylated by METTL3. Increased levels of mutant p53(R273H) cause increased drug resistance, which can be regulated through Gb3 and cSrc/β-catenin. Created with BioRender.com.

As previously demonstrated, the m^6^A epitranscriptome can influence p53 expression and function via m^6^A writers and readers, and p53 can also manipulate the m^6^A epitranscriptomic network in certain contexts. Investigations by Xu *et al.* have additionally explored the relationship between mutant p53 and the m^6^A epitranscriptomic network, and suggested that mutant p53 orchestrates neoplastic transformation through m^6^A epitranscriptome manipulation (71). By using Li–Fraumeni syndrome- (LFS; a rare inherited genetic disorder with germline *TP53* mutation) derived induced pluripotent stem cells (iPSCs) (72–74), they observed aberrant proliferation and anchorage-independent growth in LFS-derived astrocytes and organoids, which are hallmark features of cancer. They employed multi-layered analysis to delineate a dedicated regulatory complex, named the mutant p53–SVIL–MLL1 protein complex, formed by mutant p53. Interestingly, mutant p53 but not wild-type p53 interacted with the actin-binding protein SVIL and recruited H3K4me3 methyltransferase MLL1 to the *YTHDF2* promoter to activate *YTHDF2* transcription. Inhibition of the epigenetic regulators SVIL and MLL1, either genetically [short hairpin RNA (shRNA)] or pharmacologically (MLL1 inhibitor), reduced YTHDF2 expression and suppressed oncogenic transformation of LFS astrocytes and p53-mutant glioma. The increased expression of YTHDF2 and consequential rewiring of the epitranscriptomic network contribute to the tumorigenic potential of LFS cells by suppressing a panel of tumor suppressor genes, including CDKN2B. This suppression cooperates with mutant p53 to facilitate tumor initiation. Importantly, loss of CDKN2B is one of the most common genomic alterations in glioblastoma multiforme (75), which leads to CDK4/6-mediated RB1 inactivation (Figure 4A and B) (76). Their findings indicate that the mutant p53-manipulated shift in the epitranscriptomic network triggers a wide range of downstream effects, driving tumor initiation and determining tumorigenic cell fate.

### Modulating mutant p53 expression and drug resistance by the m^6^A methyltransferase complex

While DNA sequences dictate pre-mRNA sequences, additional RNA processing, including pre-mRNA splicing and RNA methylation, intricately regulates post-transcriptional protein expression (77). Unlike wild-type p53, mutant p53 possesses a distinct mRNA sequence that offers unique regulatory mechanisms, such as m^6^A modification. Uddin *et al.* have explored the dynamic relationship between m^6^A methylation and drug resistance in cancer cells carrying the p53(R273H) mutation, one of the hot-spot mutations which is found in ∼3.1% of all mutant p53-associated cancers (78). This p53(R273H) mutation is closely linked with multidrug resistance since cells with heterozygous mutations display heightened resistance to various anticancer drugs compared with those with wild-type p53. This mutation at codon 273 (G to A) facilitates m^6^A modification by METTL3 which consequently enhances mutant protein expression. Glycosphingolipids, particularly globotriaosylceramide (Gb3), were discovered to regulate METTL3 expression through cSrc/β-catenin signaling, fostering m^6^A methylation of *p53(R273H)* mRNA and influencing p53(R273H) expression and drug resistance (Figure 4C). Targeting these pathways, especially through METTL3, offers promising therapeutic strategies to combat drug resistance in cancer cells carrying this mutation.

## Conclusions and further perspectives

The above research encapsulates the significance of cross-talk between m^6^A epitranscriptomic regulation and the p53 signaling pathway, which are both essential processes for cellular function. The p53 tumor suppressor, an important regulator for DNA damage, hypoxia and nutrient deprivation, as well as signaling pathways such as apoptosis, cell cycle progression and senescence, is frequently dysregulated in cancers. Mutations in the p53 gene, found in up to 50% of all cancers (79), not only impair its tumor-suppressive function but also confer oncogenic properties, driving cancer initiation and progression across a wide range of tumor types. Similarly, the importance of m^6^A modification in RNA metabolism has garnered increasing attention in recent years. As the most prevalent post-transcriptional modification in eukaryotic mRNA, m^6^A modification regulates various aspects of RNA metabolism and cellular function. The dynamic process of m^6^A modification involves a complex interplay of writer, reader and eraser enzymes, collectively shaping the transcriptome and influencing gene expression programs essential for maintaining cellular homeostasis.

Although p53 is the most commonly mutated gene in human cancers, ∼50% of cancers still harbor wild-type p53. However, the tumor-suppressing function of p53 signaling in these p53 wild-type tumors is often impaired. Since m^6^A regulators play a critical role in regulating p53 signaling, the cancer-associated dysregulated m^6^A epitranscriptomic network indeed influences p53 function. Importantly, dysregulation of the epitranscriptome—caused by mutations, inactivation or alterations in m^6^A writers, erasers or readers—is detected in human cancers and correlates with patient prognosis (80–83). For instance, the METTL14 R298P mutation associated with cancer alters RNA methylation specificity and reduces methylation activity on canonical mRNA targets without affecting the global m^6^A regulation by METTL3/14, thereby contributing to cancer malignancy (84), implying that the function of p53 is probably altered by the METTL14 mutation. Cancer-associated alterations in m^6^A regulators can have a direct impact on gene expression, splicing, mRNA stability, proteolysis and the interactome of the p53 network, thereby hampering p53 function and signaling in p53 wild-type cancer, which leads to the inactivation of p53-mediated tumor suppression.

The cross-talk between p53 and m^6^A modification adds another layer of complexity to cellular regulatory networks. Recent research has uncovered distinctive interactions between p53 and the m^6^A epitranscriptomic network, influencing the function of both pathways. A recent breakthrough has linked p53 with METTL3, the catalytic subunit of the m^6^A methyltransferase complex. METTL3 not only regulates p53 through m^6^A modifications in both catalytically dependent and independent manners in response to DNA damage but also bolsters p53-mediated tumor suppression in specific cancer contexts. Furthermore, the methyltransferase complex exerts influence on the p53 signaling cascade by modulating the activity of p53 ubiquitin ligases FBXO43 and MDM2, which are pivotal for p53 degradation. Several m^6^A readers, such as YTHDF2 and IGFBP3 (85), have been implicated in regulating the p53 pathway across a spectrum of cancers. Although YTHDF2 has been widely recognized for its oncogenic potential in degrading *p53* mRNA, making it a promising target for cancer therapy, the other YTHDF proteins should not be overlooked. A recent study, however, has shown that YTHDF1/2/3 bind to the same m^6^A sites and primarily function in promoting mRNA degradation (15). This new evidence implies that YTHDF1 and YTHDF3 could also play a role in regulating p53 signaling. The shared functions of YTHDF1–YTHDF3 highlight the potential importance of targeting all three isoforms in cancer therapy, which is worth considering for future drug development. Additionally, proteins integral to the epitranscriptomic complex, such as METTL14 and ALKBH5, can be transcriptionally activated by p53 in certain scenarios. These studies highlight the intricate interactions between p53 and m^6^A modification. Dysregulation of the m^6^A epitranscriptome, whether through aberrant expression of m^6^A enzymes or altered p53–m^6^A epitranscriptomic interactions, can promote oncogenic transformation, tumor progression and therapy resistance. Insights gained from elucidating the molecular mechanisms underlying these interactions hold promise for the development of novel therapeutic strategies targeting the p53/m^6^A epitranscriptomic axis in cancer treatment.

While the interplay between p53 and m^6^A modification holds promising implications in cancer biology, several limitations, inconsistencies and challenges necessitate consideration in future research endeavors. Firstly, the complex regulatory networks governing p53 function and m^6^A modification remain incompletely understood, requiring further investigation to unravel their intricacies. Secondly, the same m^6^A molecule may have diverse and controversial roles in regulating p53 signaling. For example, a study by the Attardi group suggested that METTL3 positively regulates p53 function both catalytically dependently and catalytically independently of its activity. In contrast, a study by the Gong group indicated that METTL3 negatively regulates p53 by stabilizing MDM2. This discrepancy could be due to the heterogeneity observed in cancer cells, including varying mutational landscapes and differential expression patterns of m^6^A regulators. Cancer-associated mutations [e.g. *METTL14* R298P mutation (84)] and changes in gene expression of m^6^A regulators can alter their substrate specificities and m^6^A methylation patterns, affecting how m^6^A-modified mRNA is recognized by the reader proteins, with some stabilizing mRNA and others destabilizing it. Such complexities pose challenges for therapeutically targeting the p53/m^6^A epitranscriptomic axis. Moreover, the context-dependent effects of p53 mutations and m^6^A epitranscriptomic dysregulation in different cancer types underscores the need for comprehensive characterization of these interactions across a multitude of cellular contexts. Addressing these limitations will be essential for fully harnessing the therapeutic potential of targeting the p53/m^6^A epitranscriptomic axis in cancer therapies.

## Data Availability

The data presented in this article are available in the references provided.
